# The crystal structure of PD1, a *Haemophilus* surface fibril domain

**DOI:** 10.1107/S2053230X17001406

**Published:** 2017-01-31

**Authors:** Jack Wright, Maren Thomsen, Robert Kolodziejczyk, Joshua Ridley, Jessica Sinclair, Glenn Carrington, Birendra Singh, Kristian Riesbeck, Adrian Goldman

**Affiliations:** a Astbury Centre for Structural Molecular Biology, School of Biomedical Science, University of Leeds, Leeds LS2 9JT, England; b Pure Biologics Ltd, Dunska 11, 54-427 Wroclaw, Poland; c Department of Clinical Microbiology, Lund University, Jan Waldenströms gata 59, SE-205 02 Malmö, Sweden; d Division of Biochemistry, University of Helsinki, FIN-00014 Helsinki, Finland

**Keywords:** Hsf putative domain 1, trimeric autotransporter, *Haemophilus influenzae*, adhesin, cell adhesion, *Haemophilus* surface fibril

## Abstract

The *Haemophilus* surface fibril (Hsf) is an unusually large trimeric autotransporter adhesin (TAA) expressed by the most virulent strains of *H. influenzae*. Hsf is known to mediate adhesion between pathogen and host, allowing the establishment of potentially deadly diseases such as epiglottitis, meningitis and pneumonia. While recent research has suggested that this TAA might adopt a novel ‘hairpin-like’ architecture, the characterization of Hsf has been limited to *in silico* modelling and electron micrographs, with no high-resolution structural data available. Here, the crystal structure of Hsf putative domain 1 (PD1) is reported at 3.3 Å resolution. The structure corrects the previous domain annotation by revealing the presence of an unexpected N-terminal TrpRing domain. PD1 represents the first Hsf domain to be solved, and thus paves the way for further research on the ‘hairpin-like’ hypothesis.

## Abstract 2

The crystal structure of PD1 from the *Haemophilus* surface fibril was determined at a resolution of 3.3 Å, revealing a novel domain arrangement.

## 1. Introduction   

*Haemophilus influenzae* is a Gram-negative, facultative anaerobic bacterium that commonly causes upper respiratory tract infections, pneumonia and acute meningitis (Danovaro-Holliday *et al.*, 2008 ▸; Murphy *et al.*, 2009 ▸). Different strains of *H. influenzae* are either encapsulated or unencapsulated, with the former subdivided into serotypes a–f and the latter described as nontypeable (Barenkamp & St Geme, 1996 ▸). *H. influenzae* infection is established by adherence of the pathogen to the host epithelial cell linings and various extracellular matrix (ECM) proteins (*e.g.* vitronectin), in a process mediated by many pilus and nonpilus adhesive factors (Cotter *et al.*, 2005 ▸; Virkola *et al.*, 2000 ▸; Hallström *et al.*, 2006 ▸). Adhesion allows the bacterium to avoid clearance by the host, and facilitates the establishment of a deep-seated infection *via* numerous virulence mechanisms. While all strains of *H. influenzae* are pathogenic, it is the virulent type b (Hib) that, before the introduction of an effective vaccine in the 1990s, accounted for the greatest rates of patient morbidity and mortality. One such virulence factor utilized by Hib is the *Haemophilus* surface fibril (Hsf), a trimeric autotransporter adhesin (TAA) protein that shares significant homology with another, better-characterized *H. influenzae* TAA known as Hia (Cotter *et al.*, 2005 ▸; Singh *et al.*, 2015 ▸).

TAAs, which are part of the type V family of secreted proteins, have three major types of domains arranged in a linear fibril ‘lollipop’ structure. Head and stalk domains are interspersed from the N-terminus in the extracellular region. Head domains, which are formed from β-sheets with either transversal architectures, such as the YadA-like (YIhead) domains (Nummelin *et al.*, 2004 ▸), or interleaved architectures, such as the tryptophan-ring (TrpRing) domains (Szczesny *et al.*, 2008 ▸), typically mediate the adhesive activity of the proteins. The stalk forms a trimeric coiled-coil structure, with periodicity varying from heptads to pentadecads depending on the degree and direction of supercoiling (Hernandez Alvarez *et al.*, 2010 ▸). Finally, the C-terminal translocator domain is a trimeric β-barrel, with each subunit contributing one amphipathic α-helix plus four β-sheets (Meng *et al.*, 2008 ▸). This domain is responsible for the translocation of the remainder of the protein through the membrane and is found in all TAAs (Lehr *et al.*, 2010 ▸). The highly conserved nature of this domain is in contrast to the diversity observed in the TAA stalk and head domains.

Recent studies have suggested that Hsf has an apparently novel ‘hairpin-like’ structure, based on EM images (Singh *et al.*, 2015 ▸). In their shared regions, Hia and Hsf have 72% sequence identity (Hia161–1098 and Hsf1484–2413; Supplementary Fig. S1), but full-length trimeric Hsf (∼750 kDa) is more than double the size of Hia (∼340 kDa). The two binding domains of Hia (HiaBD1 and HiaBD2) have also been identified in Hsf (Laarmann *et al.*, 2002 ▸); unlike Hia, however, Hsf has an additional binding domain (HsfBD3) and three putative domains, the structure and function of which are unknown. Moreover, a limited *in silico* approach to modelling the domains of Hsf revealed that it is likely to be a linear TAA of ∼200 nm in length (Singh *et al.*, 2015 ▸). Despite this, electron micrographs of Hsf expressed in *H. influenzae* RM804 appeared to show Hsf not as a linear TAA but as a double-folded hairpin-loop structure. Mapping of the domain arrangement suggested that the N-terminus of Hsf is located close to the membrane, consistent with the ‘hairpin-like’ hypothesis.

In addition to its adhesive function, Hsf has been shown to bind the complement inhibitor vitronectin (Vn): the inter­action has been mapped to HsfBD2 and the C-terminal Vn residues 352–374 (Hallström *et al.*, 2006 ▸; Singh *et al.*, 2014 ▸). Acquisition of this glycoprotein, which is found in both serum and the ECM, allows *H. influenzae* to evade the complement system and adhere better to the epithelial surface, augmenting bacterial virulence. This may partly explain why, in contrast to Hia, Hsf is expressed in the most virulent, typeable strains of *H. influenzae*.

Here, we report the crystal structure of a Hsf putative domain, PD1. This structure reveals a novel domain arrangement for PD1, N-TrpRing:KG:TrpRing-C, and hence replaces the domain architecture previously described by *in silico* sequence analysis. This work constitutes an ongoing effort to determine the full-length structure of Hsf in order to determine whether this TAA adopts the hypothesized novel ‘hairpin-like’ structure (Singh *et al.*, 2015 ▸).

## 2. Materials and methods   

### 2.1. Macromolecule production   

#### 2.1.1. PD1-GCN4   

**Figure 1:**
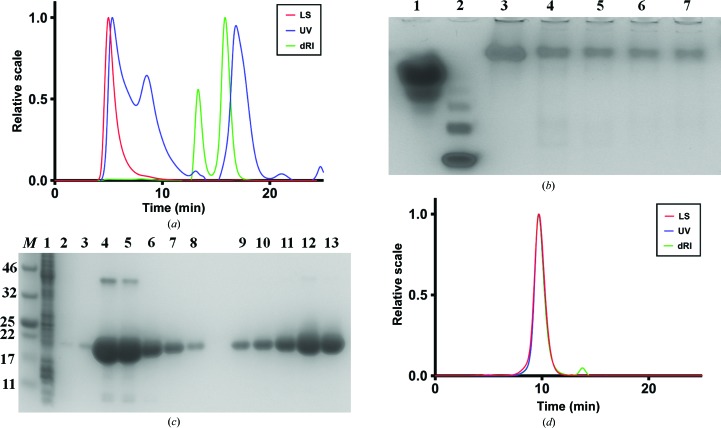
SDS–PAGE, native PAGE and SEC-MALLS demonstrating that the PD1 domains cause irreversible aggregation. (*a*) SEC-MALLS chromatogram of IMAC-purified PD1-GCN4. The degree of aggregation, as observed by the LS peak at the void volume (∼5 min), the multiple dRI and UV peaks, and an incorrect molecular weight, demonstrated that the purified protein was not amenable to crystallization. (*b*) Native PAGE of PD1-GCN4 in the presence of increasing concentrations of urea. Increasing the urea concentration had no effect on migration and hence no effect on aggregation. Lane 1, carbonic anhydrase; lanes 3–7, PD1-GCN4 in urea at varying (0, 0.5, 1, 2 and 4 *M*) concentrations. (*c*) SDS–PAGE of HsfPD1 purified by IMAC (lanes 1–8) and SEC (lanes 9–13). High levels of expression were evident (lanes 4, 5 and 6) after the proteins were separated on a gradient gel (4–20%) and visualized with Coomassie Blue. Lane *M*, molecular-weight marker (labelled in kDa); lane 1, unbound; lanes 2–3 and 8, wash; lanes 4–7, IMAC elution fractions; lanes 9–13, SEC fractions. (*d*) SEC-MALLS chromatogram of IMAC- and SEC-purified HsfPD1 [the peak corresponds to one SEC fraction, lane 12 in the SDS–PAGE gel in (*c*)]. Alignment of the LS, UV and dRI peaks, and a correct molecular weight, confirmed the presence of trimeric, non-aggregating protein.

The Hsf domain PD1 was cloned between two GCN4 anchor proteins. GCN4 is a well characterized yeast transcription factor that forms a coiled-coil dimer in its native state. However, mutagenesis of specific residues in its hydrophobic core allows GCN4 to adopt various oligomeric states. Owing to this, variations of GCN4 are often used as partners for fusion proteins to facilitate stable oligomerization. In this case, the idea was to add a well characterized trimer-forming variant of GCN4 to both the N- and C-terminus to facilitate the stable trimerization of HsfPD1 (Hernandez Alvarez *et al.*, 2008 ▸), as successfully used by the Lupas group in a number of structures (Hartmann *et al.*, 2012 ▸; Koiwai *et al.*, 2016 ▸). This fusion protein, PD1-GCN4, was expressed from a pIBA-PD1-GCN4tri-His_6_ plasmid generated using restriction-free (RF) cloning. The *PD1* gene was amplified by polymerase chain reaction from a pET-16b-*hsf* ^1–2414^ plasmid. The primers were designed to generate a ‘megaprimer’ containing the *PD1* gene with complementary overhangs to the destination vector, pIBA-GCN4tri-His_6_ (Supplementary Table S1). pIBA-GCN4tri-His_6_ was linearized by restriction digestion with XhoI (New England Biolabs) and used as a template in a secondary round of PCR to insert the *PD1* gene (contained within the ‘megaprimer’) into the plasmid. Expression of PD1-GCN4 was induced at an OD_600_ of 0.6 by the addition of anhydrotetracycline hydrochloride to a final concentration of 8.6 µ*M* for 4 h. The cells were collected by centrifugation (2000*g* for 10 min at 277 K), stored at 193 K overnight and resuspended in buffer *A* consisting of 50 m*M* NaH_2_PO_4_, 500 m*M* NaCl pH 8.0. The cells were lysed by sonication and supernatants were collected by centrifugation (16 000*g* for 10 min at 277 K). The protein was purified by immobilized metal ion-affinity chromatography (IMAC). The cleared supernatant containing PD1-GCN4 was applied onto a Ni–NTA agarose column (GE Healthcare) previously equilibrated with buffer A (2 × 6 ml; three column volumes) and allowed to bind for 1 h with agitation. Proteins were eluted in buffer *B* consisting of 50 m*M* NaH_2_PO_4_, 500 m*M* NaCl, 10% glycerol, 300 m*M* imidazole pH 8.0. The quality of the purified protein was assessed by size-exclusion chromatography coupled to a multi-angle laser light scattering (SEC-MALLS) apparatus (Fig. 1 ▸ *a*). SEC-MALLS was carried out using a Superdex 200 5/150 column pre-equilibrated with buffer *C* consisting of 50 m*M* Tris, 500 m*M* NaCl, 10% glycerol pH 8.0 at a flow rate of 0.2 ml min^−1^ and was detected using a DAWN 8+ multi-angle light-scattering (LS) detector, an Optilab T-rEX differential refractive-index (dRI) detector and a UV-absorbance (UV) detector (Wyatt).

To prevent the aggregation of PD1-GCN4 (demonstrated by SEC-MALLS), the purification was repeated in the presence of increasing concentrations of urea. The protein was expressed and the cells were lysed as above. Subsequently, the cleared supernatant was applied onto Ni–NTA agarose resin (2 ml) and allowed to bind for 1 h with agitation. Purification was performed in batch mode. The resin was washed with buffer *A* (2 × 6 ml; three column volumes) and then divided into five equal volumes for elution of protein in different buffers: buffer *B* containing 0, 0.5, 1, 2 and 4 *M* urea. The protein was eluted and fractions were collected for native PAGE analysis (Fig. 1 ▸ *b*).

#### 2.1.2. PD1   

Owing to aggregation problems with PD1-GCN4, we also expressed PD1 from a pET28-PD1-His_6_ plasmid generated using restriction-free (RF) cloning in the same way as PD1-GCN4 (§[Sec sec2.1.1]2.1.1). The *PD1* gene was amplified by PCR from the pIBA-PD1-GCN4tri-His_6_ plasmid using primers capable of producing a ‘megaprimer’. The pET-28-Tcfa-His_6_ destination vector was linearized by restriction digestion with XhoI and NcoI (New England Biolabs) to remove the *tcfA* gene (while retaining the His_6_ tag). This plasmid backbone was the template for a secondary round of PCR, utilizing the ‘megaprimer’, to insert the *PD1* gene into the plasmid (Table 1 ▸). Expression of PD1 was induced at an OD_600_ of 0.6 by the addition of isopropyl β-d-1-thiogalactopyranoside (IPTG) to a final concentration of 0.5 m*M* for 4 h. The cells were collected and stored as before (§[Sec sec2.1.1]2.1.1) and resuspended in buffer *C* consisting of 50 m*M* Tris, 150 m*M* NaCl pH 8.0. The cells were lysed by sonication and supernatants were collected by centrifugation (16 000*g* for 10 min at 277 K). The protein was purified *via* IMAC on a Ni–NTA agarose column previously equilibrated with buffer *C* (2 × 6 ml; three column volumes) and allowed to bind for 1 h with agitation. Proteins were eluted in buffer *D* consisting of 50 m*M* Tris, 150 m*M* NaCl, 300 m*M* imidazole pH 8.0, and the pooled fractions were concentrated to 500 µl. Further purification was carried out by size-exclusion chromatography (SEC) on a Superdex 200 10/300 column pre-equilibrated with buffer *E* consisting of 50 m*M* Tris, 600 m*M* NaCl pH 8.0 and eluting imidazole-free protein at a flow rate of 0.2 ml min^−1^. The purified fractions were then pooled and concentrated to 15 mg ml^−1^ for crystallization. The quality of the purified protein was assessed prior to crystallization by SDS–PAGE and SEC-MALLS, carried out as described above, using buffer *C* (Fig. 1*c* and 1*d* ▸).

### 2.2. Crystallization   

Initial PD1 crystals were obtained using the Wizard Classic 3 and 4 crystallization screens (Molecular Dimensions) using the following conditions: protein concentration 15 mg ml^−1^, 1 *M* LiCl, 0.1 *M* sodium citrate pH 4, 20%(*w*/*v*) polyethylene glycol (PEG) 6000. Crystallization was performed at 293 K using the sitting-drop vapour-diffusion method, in which 100 nl protein solution was mixed with an equal volume of reservoir solution. Drops were set up using a Formulatrix NT8 crystallization robot. Since the initial crystals diffracted poorly, further crystallization optimization of PD1 was performed. The best diffracting crystals grew from 0.75 *M* LiCl, 0.1 *M* sodium citrate pH 3.9, 17.6%(*w*/*v*) PEG 6000 (Table 2 ▸). Owing to the presence of PEG, this solution already had cryoprotectant properties and thus the crystals were flash-cooled directly in liquid nitrogen for data collection.

### 2.3. Data collection, processing and structure determination   

Data were collected on beamline I03 at Diamond Light Source (DLS). Although radiation damage restricted the data set to the first 1100 images, the crystals diffracted to 3.3 Å resolution (Table 3 ▸). Indexing and integration were performed using *XDS* (Kabsch, 2010 ▸), while scaling and merging statistics were calculated using *AIMLESS* (Evans & Murshudov, 2013 ▸). The structure of PD1 was solved by molecular replacement (MR) with *Phaser* (McCoy *et al.*, 2007 ▸), using the non-adhesive domain of Hia307–422 (PDB entry 3emi; Meng *et al.*, 2008 ▸; 72.3% sequence identity) as the search model. *Phaser* found one unique solution in space group *C*2, with nine monomers in the asymmetric unit forming three trimers. The translation-function *Z*-score (TFZ) of 39.95 and log-likelihood gain (LLG) of 2010 indicated a correct MR solution. Refinement was carried out with *PHENIX* (Adams *et al.*, 2010 ▸), using secondary-structure and noncrystallographic symmetry torsion restraints, and the structure was refined to an *R* factor of 0.296 (Table 4 ▸).

## 3. Results and discussion   

### 3.1. Purification of PD1-GCN4 and PD1   

Initial efforts to purify PD1 utilizing GCN4 anchors (PD1-GCN4; Hernandez Alvarez *et al.*, 2008 ▸; Deiss *et al.*, 2014 ▸) were not successful owing to complete protein aggregation (Fig. 1 ▸ *a*). This was unexpected, as 18 crystal structures of TAA domains have already been solved using this method. We thought that the aggregation might be owing to hydrophobic interactions between the head domains and/or owing to improper folding of these domains arising from their flanking by the GCN4. We therefore decided to purify the protein in the presence of increasing concentrations of urea (0.5–4 *M*) to prevent aggregation and to use native PAGE to assess the level of aggregation (Fig. 1 ▸ *b*). However, this method was unsuccessful in reducing aggregation, as the protein still did not migrate as expected in the gel, suggesting that the GCN4 anchors cause extensive misfolding and not just a small amount of reversible aggregation. We therefore decided to remove the GCN4 anchors. The construct lacking GCN4 (§[Sec sec2.1.2]2.1.2) yielded protein that was amenable to crystallization. SEC-MALLS showed that the purified PD1 was trimeric (Fig. 1*d* ▸); the molecular weight of the peak in the chromatogram was 45.6 kDa, as determined from the UV, LS and dRI signals using the *ASTRA* software (Wyatt). This is within experimental error (±5%) of the expected molecular weight of 47.8 kDa. It was clear from the chromatogram that no aggregates were present, and this construct yielded diffracting crystals (Supplementary Fig. S4).

### 3.2. Structure of PD1   

In our effort to characterize the full-length Hsf protein, we determined the crystal structure of trimeric PD1 at a resolution of 3.3 Å, thus providing the first insight into the molecular arrangement of Hsf to date. HsfPD1 crystallized in the monoclinic space group *C*2 (Table 1 ▸), with nine monomers in the asymmetric unit. The crystals had an estimated solvent content of 56.5%, with a Matthews coefficient (*V*_M_) of 2.83 Å^3^ Da^−1^. The number of residues identified in the density of each monomer varied between 129 and 133 residues. The missing loops in the monomers are owing to poor electron density. Typical density is presented in Supplementary Fig. S3.

**Figure 2:**
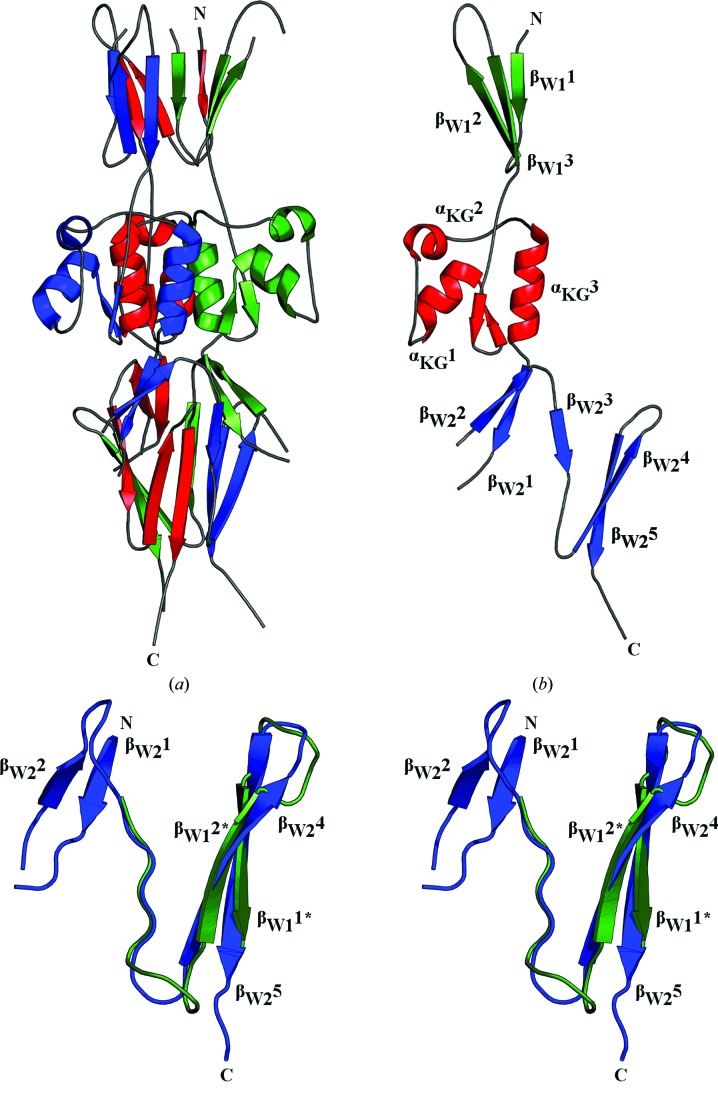
The crystal structure of HsfPD1 at 3.3 Å resolution. (*a*) HsfPD1 showing a trimeric architecture (three monomer subunits; blue, red and green). (*b*) One HsfPD1 subunit with labelled secondary structure showing a novel domain arrangement: N-TrpRing:KG:TrpRing-C (proposed N-terminal TrpRing domain, green; KG domain, red; C-terminal TrpRing, blue). (*c*) 29 N-terminal HsfPD1 residues superimpose on 29 C-terminal HsfPD1 residues with an r.m.s.d. of 1.16 Å for the backbone (29 N-terminal residues, green; original C-terminal TrpRing, blue). β_W1_1–3, proposed novel TrpRing; α_KG_1–3, KG-domain helices [KG-domain β-strand labels omitted for clarity in (*b*)]; β_W2_1–5, original TrpRing [β_W2_3 omitted for clarity in (*c*)].

The individual monomers of HsfPD1 are comprised of three distinct domains that fold to form well characterized TAA domains. A proposed N-terminal TrpRing domain, a KG domain and a C-terminal TrpRing domain are seen in each PD1 monomer (Fig. 2 ▸ *b*). The N-terminus of HsfPD1 spans 29 amino acids participating in the unexpected formation of three β-sheets, β_W1_1, β_W1_2 and β_W1_3 (where ‘W’ represents tryptophan), which share considerable structural homology with the C-terminal TrpRing domain. Although the sequence identity between these two regions is low (31%; Supplementary Fig. S2), a structural alignment of the 29 N- and C-terminal residues from one HsfPD1 monomer (Fig. 2 ▸ *c*) confirms that the N-terminal region is indeed a TrpRing domain. The KG domain is composed of two β-strands, β_KG_1 and β_KG_2, as well as three α-helices, α_KG_1, α_KG_2 and α_KG_3. The C-terminal TrpRing is composed of five β-strands: β_W2_1, β_W2_2, β_W2_3, β_W2_4 and β_W2_5. All domains participate in extensive intertwining, where the C-terminal α-helices (α_KG_3) from each monomer come together to create the central core of the trimer interface. The KG and C-terminal TrpRing domains were easily identified by simple structural observation and comparison with other TAAs (Meng *et al.*, 2008 ▸).

The TrpRing domains of TAAs are so named for the highly conserved tryptophan residue that resides at the beginning of the first β-strand. Owing to the structural homology between β-sheets β_W1_1, β_W1_2, β_W1_3 and the C-terminal TrpRing, we further analysed the full-length Hsf sequence and identified a tryptophan residue 27 residues upstream of our HsfPD1 N-terminus. Since our construct contained only 29 N-terminal residues upstream of the KG domain, and as TrpRing domains typically consist of ∼55 amino acids, the structural evidence suggests that our N-terminal β-strands constitute the latter half of a TrpRing domain. Additionally, the interleaved nature of this proposed TrpRing domain and the fact that its N- and C-termini lie close to the trimer axis support this hypothesis. Prior to the solution of this structure, sequence analysis of full-length Hsf resulted in the annotation of HsfPD1 as a duplicate domain: N-KG:TrpRing-C (Singh *et al.*, 2015 ▸). However, our crystal structure indicates a novel triplicate domain arrangement for HsfPD1, N-TrpRing:KG:TrpRing-C, an arrangement that is likely to extend to all Hsf putative domains.

### 3.3. Comparison of HsfPD1 with HiaBD1 and Hia307–422   

**Figure 3:**
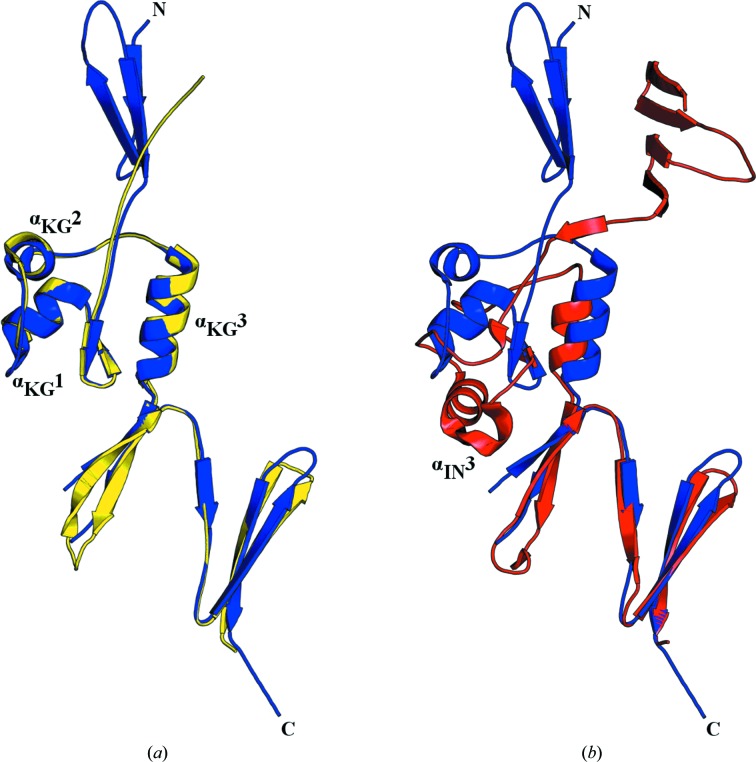
Superposition of HsfPD1 with Hia307–422 and HiaBD1. (*a*) HsfPD1 aligns with Hia307–422 with an r.m.s.d. of 0.784 Å for the backbone, demonstrating strong homology (HsfPD1, blue; Hia307–422, yellow). (*b*) HsfPD1 aligns with HiaBD1 with an r.m.s.d. of 0.969 Å for the backbone, clearly showing the lack of α_IN_3 in HsfPD1. HsfPD1, blue; HiaBD1, red.

Hsf and Hia are remarkably similar in their domain arrangement, as both possess adhesive domains (BDs) and domains of unknown function (PDs). Whilst BD domains have contiguous Neck-TrpRing architecture, the PD domains have a KG domain instead of the Neck domain. The adhesive activity of the BD domains in Hia results from the formation of an acidic pocket created by residues Asp618 and Ala620 of α_IN_3, along with Val656 of the C-terminal TrpRing (Yeo *et al.*, 2004 ▸; Cotter *et al.*, 2005 ▸). The substitution of the Neck domain for KG domains abrogates the adhesive activity of PDs owing to the lack of an equivalent α-helix in KG to that of α_IN_3 from the Neck domain. Indeed, a superposition of HsfPD1 with the non-adhesive head domain of Hia (PDB entry 3emi; Meng *et al.*, 2008 ▸) shows strong structural similarity (Fig. 3 ▸ *a*). In contrast, although a superposition of HsfPD1 with HiaBD1 (PDB entry 1s7m; Yeo *et al.*, 2004 ▸) reveals modest structural similarity, the acidic pocket created by α_IN_3 is clearly missing in HsfPD1 (Fig. 3 ▸ *b*), suggesting that HsfPD1 is indeed a non-adhesive domain.

### 3.4. Evolution of putative domains in Hia and Hsf   

Owing to the high sequence identity between the shared regions of Hia and Hsf (Supplementary Fig. S1), we predict that, had the N-terminus of the Hia307–422 construct been extended by ∼40 residues, the same N-TrpRing:KG:TrpRing-C arrangement would have been observed. Moreover, this triplicate arrangement indicates an evolutionary link between BD and PD domains, in that BD domains are indeed triplicates, *i.e.* N-TrpRing:Neck:TrpRing-C, and Hsf is approximately double the length of Hia. Thus, PD domains may have evolved *via* the duplication of BD domains or *vice versa*. This duplication certainly contributes to the overall length of Hsf, and whilst it is consistently reported that the PD domains are of unknown function, one implication of this evolution is that the additional length created by these domains conveys a survival advantage on those strains of *H. influenzae* that express Hsf. This perhaps explains why Hsf is expressed by all typeable strains of *H. influenzae* (*e.g.* type b; Hib) whilst Hia is not, *i.e.* it is long enough to extend beyond the bacterial lipopolysaccharide layer and thus bind to complement regulators and ECM molecules to evade attack by the host.

## 4. Conclusion   

Although HsfPD1 is in many respects a typical TAA domain, the novel domain arrangement (N-TrpRing:KG:TrpRing-C), revealing the N-terminal TrpRing domain, demonstrates the necessity of structural characterization of such proteins, as opposed to sequence analysis alone. This arrangement yielded insights into the evolution of PD domains, supporting the divergent nature of TAAs, and supersedes the previous domain annotation. Furthermore, the structure of HsfPD1 will contribute to the understanding and determination of the hypothesized ‘hairpin-like’ structure of Hsf. Inclusion of the N-terminal TrpRing domain in computer models may help to refine them. This combination may reveal unique protein–protein interactions between antiparallel PD and BD domains, generating exciting insights into the structure of TAAs, should this novel hypothesis be true.

## Supplementary Material

PDB reference: Hsf 1608–1749 putative domain 1, 5lnl

Supplementary Tables and Figures.. DOI: 10.1107/S2053230X17001406/no5112sup1.pdf

## Figures and Tables

**Table 1:** PD1 production information

Source organism	*H. influenzae*
DNA source	pIBA-PD1-GCN4tri-His_6_
Forward primer	GTTTAACTTTAAGAAGGAGATATACCATGGACTTTGTTAGTGGAG
Reverse primer	GTGGTGGTGGTGCTCGAGGTCTAGTTTTAAGCCATCAGCCAC
Cloning vector	pET-28
Expression vector	pET-28
Expression host	*Escherichia coli* BL21*
Complete amino-acid sequence of the construct produced	MDFVSGDKDTTSVTVESKDNGKRTEVKIGAKTSVIKDHNGKLFTGKELKDANNNGVTVTETDGKDEGNGLVTAKAVIDAVNKAGWRVKTTGANGQNDDFATVASGTNVTFADGNGTTAEVTKANDGSITVKYNVKVADGLKLD

**Table 2:** Crystallization

Method	Sitting-drop vapour diffusion
Plate type	96-well
Temperature (K)	293
Protein concentration (mg ml^−1^)	15
Buffer composition of protein solution	50 m*M* Tris, 600 m*M* NaCl pH 8.0
Composition of reservoir solution	0.75 *M* LiCl, 0.1 *M* sodium citrate pH 3.9, 17.6%(*w*/*v*) PEG 6000
Volume and ratio of drop (nl)	200, 1:1
Volume of reservoir (µl)	50

**Table 3:** Data collection and processing Values in parentheses are for the outer shell.

Diffraction source	I03, DLS
Wavelength (Å)	0.9796
Temperature (K)	100
Detector	PILATUS3 6M
Crystal-to-detector distance (mm)	604
Rotation range per image (°)	0.1
Total rotation range (°)	110
Exposure time per image (s)	0.1
Space group	*C*2
*a*, *b*, *c* (Å)	128.4, 50.4, 256.8
α, β, γ (°)	90, 101.9, 90
Mosaicity (°)	0.42
Resolution range (Å)	29.6–3.3 (3.53–3.30)
Total No. of reflections	50038 (9023)
No. of unique reflections	24219 (4309)
Completeness (%)	97.7 (97.8)
Multiplicity	2.1 (2.1)
〈*I*/σ(*I*)〉	2.7 (0.9)
Half-set correlation CC_1/2_	0.962 (0.49)
*R*_merge_	0.333 (1.14)
*R*_r.i.m._	0.425 (1.39)
*R*_p.i.m_	0.286 (0.952)
Overall *B* factor from Wilson plot (Å^2^)	62.9

**Table 4:** Structure solution and refinement Values in parentheses are for the outer shell.

Resolution range (Å)	29.6–3.3 (3.53–3.30)
Completeness (%)	97.6 (97.8)
No. of reflections, working set	24110
No. of reflections, test set	1178
Final *R*_cryst_	0.296
Final *R*_free_	0.334
No. of non-H protein atoms	8073
R.m.s. deviations	
Bonds (Å)	0.002
Angles (°)	0.472
Average *B* factors (Å^2^)	58.9
Ramachandran plot	
Most favoured (%)	98.04
Outliers (%)	0.18
