# Supplementary material for: The crystal structure of PD1, a Haemophilus surface fibril domain
Source: Acta Crystallogr F Struct Biol Commun. 2017 Jan 31;73(Pt 2):101–8. doi: 10.1107/S2053230X17001406 (PMC5297931; doi:10.1107/S2053230X17001406)
Supplement: Supplementary file 1 [file f-73-00101-sup1.pdf]

Acta Cryst F STRUCTURAL BIOLOGY COMMUNICATIONS

Volume 73 (2017)

Supporting information for article:

The crystal structure of a *Haemophilus* surface fibril domain, PD1

Jack Wright, Maren Thomsen, Robert Kolodziejczyk, Joshua Ridley, Jessica Sinclair, Glenn Carrington, Birendra Singh, Kristian Reisbeck and Adrian Goldman

Table S1 Construct generation information for HsfPD1-GCN4

	PD1-GCN4
Source organism	*Haemophilus influenzae*
DNA source	pET16b-*hsf* $^{1-2414}$
Forward primer	GCGCGTATTAAAAAACTGATTGTGGACTTTGTTAGTGGAGATAAA
Reverse primer	CAATTTTATCTTCAATCTGTTTCATGTCTAGTTTTAAGCCATCAGCCAC
Cloning vector	pIBA-GCN4tri-His$_6$
Expression vector	pIBA-GCN4tri-His$_6$
Expression host	*Escherichia coli* BL21*
Complete amino acid sequence	MKQIEDKIEEILSKIYHIENEIARIKKLIVDFVSGDKDTTSVTVESKDNGKRTE VKIGAKTSVIKDHNGKLFTGKELKDANNNGVTVTETDGKDEGNGLVTAKA VIDAVNKAGWRVKTTGANGQNDDFATVASGTNVTFADGNGTTAEVTKAN DGSITVKYNVKVADGLKLDMKQIEDKIEEILSKIYHIENEIARIKKLIKLHHHH HH

```
Hia161-1098     ITFALAKDLGVKTATVSDTLTIGGGAAAGATTTPKVNVTSTTDGLKFAKDAAGANGDTTV
Hsf1484-2413    ITFALANDLSVKSATVSDKLSLG-------TNGNKVNITSDTKGLNFAKDSKTGD-DANI
                ******:**.**:*****.*::*       *.  ***:** *.**:****:  .: *:.:

Hia161-1098     HLNGIGSTLTDTLVGSPATHIDGGDQSTHY--TRAASIKDVLNAGWNIKGVKAGSTTGQS
Hsf1484-2413    HLNGIASTLTDTLLNSGATTNLGGNGITDNEKKRAASVKDVLNAGWNVRGVKPASANNQV
                *****.*******:.* **   **:  *    .****:*********:.***..*:..*

Hia161-1098     ENVDFVHTYDTVEFLSADTETTTVTVDSKENGKRTEVKIGAKTSVIKEKDGKLFTGKANK
Hsf1484-2413    ENIDFVATYDTVDFVSGDKDTTSVTVESKDNGKRTEVKIGAKTSVIKDHNGKLFTGKELK
                **:*** *****:*:*.*.:**:***:**:*****************:::*******  *

Hia161-1098     ETNKVDGANATE--DADEGKGLVTAKDVIDAVNKTGWRIKTTDANGQNGDFATVASGTNV
Hsf1484-2413    DANN-NGVTVTETDGKDEGNGLVTAKAVIDAVNKAGWRVKTTGANGQNDDFATVASGTNV
                ::*: :*...**  . ***:****** *******:***:***.*****.***********

Hia161-1098     TFASGNGTTATVTNGTDG-ITVKYDAKVGDGLKLDGDKIAADTTALTVNDGKNANNPKGK
Hsf1484-2413    TFADGNGTTAEVTKANDGSITVKYNVKVADGLKLDGDKIVADTTVLTVADGKVTAPNNG-
                ***.****** **:..** *****:.**.**********.****.*** *** :   :*

Hia161-1098     VADVASTDEKKLVTAKGLVTALNSLSWTTTAAEADGGTLD-GNASEQEVKAGDKVTFKAG
Hsf1484-2413    -------DGKKFVDASGLADALNKLSWTATAGKEGTGEVDPANSAGQEVKAGDKVTFKAG
                       * **:* *.**. ***.****:**.: . * :* .*:: **************

Hia161-1098     KNLKVKQEGANFTYSLQDALTGLTSITLGTGNNGA---KTEINKDGLTITPANG---AGA
Hsf1484-2413    DNLKIKQSGKDFTYSLKKELKDLTSVEFKDANGGTGSESTKITKDGLTITPANGAGAAGA
                .***:**.* :*****:. *..***: :  .*.*:   .*:*.***********   ***

Hia161-1098     NNANTISVTKDGISAGGQSVKNVVSGLKKFGDANFDPLTSSADNLTKQNDDAYKGLTNLD
Hsf1484-2413    NTANTISVTKDGISAGNKAVTNVVSGLKKFGDGH-TLANGTVADFEKHYDNAYKDLTNLD
                *.**************.::*.***********.:    ..:. :: *: *:***.*****

Hia161-1098     EKGTDKQTPVVADNTAATVGDLRGLGWVISADKTTGG-STEYHDQVRNANEVKFKSGNGI
Hsf1484-2413    EKGADNN-PTVADNTAATVGDLRGLGWVISADKTTGEPNQEYNAQVRNANEVKFKSGNGI
                ***:*:: *.**************************  . **: ****************

Hia161-1098     NVSGKTVNGRREITFELAKGEVVKSNEFTVKETNGKETSLVKVGDKYYSKEDIDLTTGQP
Hsf1484-2413    NVSGKTLNGTRVITFELAKGEVVKSNEFTVKNADGSETNLVKVGDMYYSKEDIDPATSKP
                ******:** * *******************:::*.**.****** ******** :*.:*

Hia161-1098     KLKDGNTVAAKYQDKGGKVVSVTDN-TEATITNKGSGYVTGNQVADAIAKSGFELGLADE
Hsf1484-2413    --MTGKT--EKYKVENGKVVSANGSKTEVTLTNKGSGYVTGNQVADAIAKSGFELGLADA
                    *:*   **: :.*****.... **.*:****************************

Hia161-1098     ADAKAAFDD--KTKALSAGTTEIVNAHDKVRFANGLNTKVSAATVESTDANGDKVTTTFV
Hsf1484-2413    AEAEKAFAESAKDKQLSKDKAETVNAHDKVRFANGLNTKVSAATVESTDANGDKVTTTFV
```

```
                          *:*: ** :  * * ** ..:* *************************************

Hia161-1098               KTDVELPLTQIYNTDANGKKITKVVKDGQTKWYELNADGTADMTKEVTLGNVDSDGKKVV
Hsf1484-2413              KTDVELPLTQIYNTDANGNKI---VKKADGKWYELNADGTAS-NKEVTLGNVDANGKKVV
                          ******************:**   **..: ***********. .*********::*****

Hia161-1098               K---DNDGKWYHAKADGTADKTKGEVSNDKVSTDEKHVVSLDPNDQSKGKGVVIDNVANG
Hsf1484-2413              KVTENGADKWYYTNADGAADKTKGEVSNDKVSTDEKHVVRLDPNNQSNGKGVVIDNVANG
                          *   :. .***:::***:********************* ****:**:************

Hia161-1098               DISATSTDAINGSQLYAVAKGVTNLAGQVNNLEGKVNKVGKRADAGTASALAASQLPQAT
Hsf1484-2413              EISATSTDAINGSQLYAVAKGVTNLAGQVNNLEGKVNKVGKRADAGTASALAASQLPQAT
                          :***********************************************************

Hia161-1098               MPGKSMVAIAGSSYQGQNGLAIGVSRISDNGKVIIRLSGTTNSQGKTGVAAGVGYQW
Hsf1484-2413              MPGKSMVAIAGSSYQGQNGLAIGVSRISDNGKVIIRLSGTTNSQGKTGVAAGVGYQW
                          *********************************************************
```

Figure S1 MUSCLE sequence alignment of shared regions in Hia (Hia161-1098) and Hsf (Hsf1484-2413), demonstrating 72% sequence identity (identical residues, red/asterisk; strongly similar residues, colon; weakly similar residues, full stop).

```
N-TERMINUS                ---DFVSGDKDTTSVTVESKDNGKRTEVKIGA
C-TERMINUS                TNVTFADGNGTTAEVT---KANDGSITVKYNV
                              *..*:  *:.**   * *.    ** ..
```

Figure S2 MUSCLE sequence alignment of 29 N- and C-terminal residues of HsfPD1 demonstrating 31% sequence identity (annotation as in Supplementary Fig. S1).

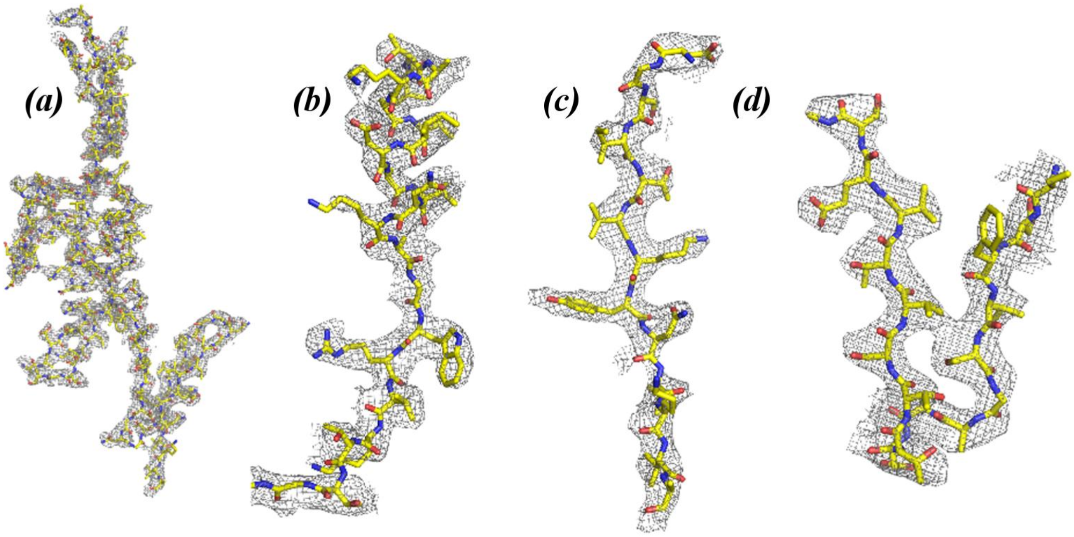

Figure S3 Electron density maps of HsfPD1 after refinement. Representative presentations of $2F_o$-F_c electron density maps of (a) overall chain D and various regions within chain D: (b) amino acids Val1677 – Ala1698, (c) C-terminus (Asp1730 – Ala1743) and (d) N-terminus (Met1607 – Ser1623). All maps are contoured at 1σ.

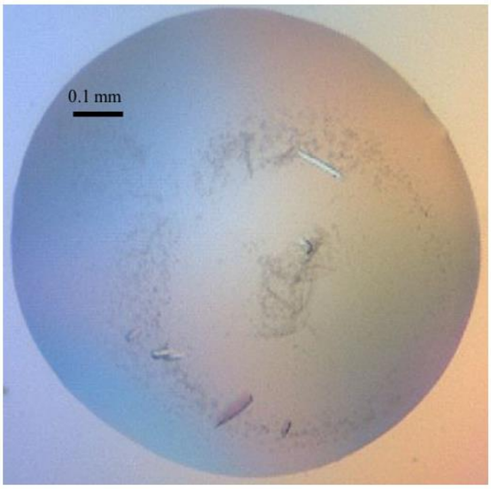

Figure S4 Crystals of PD1.
